# hnRNP K plays a protective role in TNF-α-induced apoptosis in podocytes

**DOI:** 10.1042/BSR20180288

**Published:** 2018-06-12

**Authors:** Shili Zhao, Junxia Feng, Qi Wang, Lu Tian, Yunfang Zhang, Hongyan Li

**Affiliations:** Department of Nephrology, Huadu District People’s Hospital, Southern Medical University, Guangzhou 510800, China

**Keywords:** apoptosis, c-FLIP, hnRNP K, podocyte, TNF-α

## Abstract

Apoptosis of podocytes contributes to proteinuria in many chronic kidney diseases. The cytokine, tumor necrosis factor-α (TNF-α) is thought to be involved in podocyte apoptosis, but the underlying mechanism is not understood. In our study, we established a model of TNF-α-induced apoptosis by isolating primary podocytes from mice. After exposing cells to TNF-α, we determined the expression levels of heterogeneous nuclear ribonucleoprotein K (hnRNP K) and cellular FLICE-inhibitory protein (c-FLIP) and the phosphorylation levels of glycogen synthase kinase β (GSK3β) and extracellular signal-regulated kinase (ERK). We then knocked down or overexpressed the levels of hnRNP K and observed its effects on the expressions of c-FLIP, caspase-8, caspase-3, and the phosphorylation of GSK3β and ERK. In addition, we examined the percentage of cells undergoing apoptosis and studied cell cycle distribution. We found that TNF-α induced apoptosis in podocytes and that the expressions of hnRNP K and c-FLIP were significantly decreased, whereas the phosphorylations of GSK3β and ERK were significantly increased. Both gene knockdown and overexpression of hnRPN K resulted in varied expressions/phosphorylations of c-FLIP, GSK3β, and ERK. Moreover, decreased hnRPN K expression contributed to increased levels of caspase-8 and capase-3, as well as an increase in cell apoptosis and G0/G1 arrest. In conclusion, down-regulated expression of hnRNP K by TNF-α resulted in a decrease in the expression of c-FLIP as well as increases in phosphorylated GSK3β, ERK, caspase-8, and caspase-3, and then critically contributed to the podocyte apoptosis.

## Introduction

As a highly differentiated and structurally complex organ, the kidney is composed of approximately a million clusters of looping blood vessels called glomeruli. The outer part of the glomerular basement membrane is covered with extremely ramified cells called podocytes. As blood flows through each glomerulus, water and metabolic wastes are filtered through capillary walls by the surrounding podocytes. Therefore, podocytes function as the final barrier to protein loss and are crucial in maintaining the integrity of the glomerular filtration barrier [1]. Apoptosis is a major reason for podocyte loss and sequentially results in glomerular diseases [2]. Recent studies revealed that podocyte apoptosis and loss are observed in many forms of human and experimental glomerular diseases, including minimal change disease (MCD), focal segmental glomerulosclerosis, membranous glomerulopathy, diabetes mellitus, and lupus nephritis [1]. Therefore, preventing podocyte apoptosis is an attractive therapy for treating these kidney diseases. However, the underlying mechanisms involved in podocyte apoptosis are still not fully understood.

Tumor necrosis factor-α (TNF-α) is a proinflammatory cytokine secreted by a variety of cell types, including macrophages, lymphocytes, natural killer cells, and epithelial cells. It regulates several cellular responses including proinflammatory cytokine production, cell survival, cell proliferation, and paradoxically, cell death. TNF-α has been shown to induce podocyte apoptosis [3]. Furthermore, TNF-α blockade showed protective effects on glomerulosclerosis of hypertensive rodents and on podocyte apoptosis [3]. It is widely acceptable that apoptosis induced by TNF-α is caspase-dependent [4]. Additionally, TNF-α, released by macrophages, has been shown to inhibit cAMP-RAR and ROS-p38MAPK pathway, leading to podocyte injury [5,6].

Heterogeneous nuclear ribonucleoprotein K (hnRNP K) is a ubiquitously expressed protein and belongs to the subfamily of heterogeneous ribonucleoproteins (hnRNPs). hnRNP K is involved in multiple processes including chromatin remodeling, transcription, RNA splicing, mRNA stability, and translation [7]. In addition to being associated with various physiological processes, hnRNP K participates in several human diseases. A recent study showed significantly decreased hnRNP K expression in the mice with diabetic nephropathy and suggested induced expression attenuated the nephropathy [8]. Podocyte apoptosis always occurred at the onset of diabetic nephropathy. However, there is no information on the correlation between podocyte apoptosis and hnRNP K. Accumulating evidence suggests that hnRNP K plays an important role in the development of many types of cancers, including pancreatic cancer [9]. The tumorigenic activity of hnRNP K is ascribed to its proliferative and antiapoptotic effects, which are achieved by inducing downstream antiapoptotic proteins. Furthermore, hnRNP K has been shown to be associated with cellular FLICE-inhibitory protein (c-FLIP), glycogen synthase kinase β (GSK3β), and extracellular signal-regulated kinase (ERK) [9,10].

c-FLIP is a master regulator of apoptosis-mediating factors. It suppresses apoptosis induced by TNF-α, Fas-L, and TNF-related apoptosis-inducing ligand (TRAIL). Moreover, c-FLIP shares structural homology with procaspase-8 but lacks the catalytic site. It can therefore competitively inhibit the activation of caspase-8 and act as a key suppressor of the death receptor signaling pathway.

The GSK3β and ERK are two other key regulators of apoptosis. GSK3β has been shown to exhibit dual functions as an apoptosis inhibitor or a promoter and also be involved in repair and acute injury [11]. Recent studies demonstrated that inhibition of GSK3 by celecoxib promoted the degradation of c-FLIP and death receptor-induced apoptosis, suggesting that GSK3 might stabilize c-FLIP and antagonize tumor resistance to TRAIL [12]. On the other hand, inhibition of GSK3β by lithium has been shown to have a protective role in podocytopathy [13]. ERK is involved in the activation of the extrinsic pathway by TNF-α and TRAIL, as well as in podocyte apoptosis [14].

In the present study, we investigated the expression levels of hnRNP K, c-FLIP, GSK3β, and ERK and determined their phosphorylation status in podocytes treated with a low dose of TNF-α. Furthermore, we demonstrated that knocking down or overexpressing hnRNP K–mediated podocyte apoptosis by regulating the expression of c-FLIP, and the phosphorylation of GSK3β and ERK. Our data suggest that hnRNP K could be a potential target for therapeutic intervention in proteinuric glomerulopathies.

## Materials and methods

### Isolation and cultivation of mouse podocytes

The male mice at the age of 6–8 weeks were purchased from the Central Animal Facility of Southern Medical University and used for cell isolation. All the procedures conducted on the animals were approved by the Animal Care and Use Committee of Southern Medical University. The mice were anesthetized with pentobarbital at 0.07 mg/kg, and then their kidneys were collected. This was followed by renal capsule removement in PBS containing 1% penicillin/streptomycin (Sigma–Aldrich, St Louis, MO, U.S.A.). The medullas were removed and the kidneys were minced into small pieces of 1–3 mm^3^ sized pieces, and then gently ground. The suspension was filtered through a succession of cell strainers of 80, 120, and 200 mesh. The filtrate was centrifuged at 50 ***g*** for 2 min. The pellet was resuspended with 0.1% collagenase I in RPMI 1640 and then incubated at 37°C in a shaker for 20 min. The cell suspension was then centrifuged at 100 ***g*** for 5 min. The cells were resuspended in complete culture medium (Procell, Wuhan, China). Thereafter, the cells were cultured in the complete medium on dishes precoated with polylysine at 37°C, 5% CO_2_, and the medium was refreshed every 3 days.

### Cell viability

Cell viability was detected with a CCK8 kit (KeyGen, Nanjing, China). In brief, podocytes were seeded in 96-well plates at a density of 10^4^ cells/well and grown in 100 μl complete medium overnight at 37°C with 5% CO_2_. After 24-h treatment with either control or TNF-α at the concentrations of 10, 20, 40, 80, or 160 ng/ml, the medium was refreshed and CCK8 (10 μl) was added into each well. After 2 h of incubation, absorbance was measured at 450 nm using a microplate reader (Bio–Rad Laboratories, Hercules, CA, U.S.A.). The actual absorbance value of each well = OD value of the test well – OD value of the blank. Finally, the viability curve was prepared.

### Cell transfection

The siRNA oligos targetting hnRNP K were designed and transfected into cells. In brief, the siRNA (si hnRNPK: sense 5′-UGUACGGAGAGCCUUAAUATT-3′ and siRNA negative control (NC)) were obtained from GenePharma (Shanghai, China). Transfections were performed with either 75 nM siRNA or NC, using Lipofectamine RNAiMAX (Invitrogen, CA, U.S.A.). The cDNA encoding hnRNP K was amplified by PCR and then subcloned into pcDNA3.0 vectors. Empty pcDNA3.0 vector was used as a negative control. Transfections of pcDNA 3.0 were performed with Lipofectamine 2000 reagents (Invitrogen, CA, U.S.A.), according to the manufacturer’s instructions.

### Flow cytometric analysis

After exposure to 5 ng/ml of TNF-α for 24 h, the cells were detached with 0.25% EDTA-free trypsin, and harvested for apoptosis and cell cycle analysis. After centrifugation at 1000 rpm for 5 min, the medium was discarded and the cells were resuspended in precooled PBS for apoptosis analysis. Following overnight fixing of cells in precold 70% ethanol at 4°C, they were washed twice with PBS and incubated in a binding buffer containing FITC-conjugated Annexin V and propidium iodide (PI, KeyGen, Nanjing, China) at 4°C for 30 min in the dark. Data collection was performed using BD FACSCalibur flow cytometer (BD Biosciences, CA, U.S.A.). Additionally, cells were also used in proliferation and cell cycle assays with the EdU Alexa Fluor 488 Flow Cytometry assay kit (Riobio, Guangzhou, China), according to the manufacturer’s protocol. After 24 h of treatment with 5 ng/ml of TNF-α, the cells were incubated with EdU and then washed with PBS, fixed with 0.5% buffered paraformaldehyde, permeabilized with cold acetone for 3 min and then incubated with the Alexa Fluor 488 EdU detection solution for 30 min in the dark. Some cells were subjected to proliferation assay with the flow cytometry. Other cells received an additional staining with 25 μg/ml PI (Sigma, MO, U.S.A.), and then subjected to cell cycle analysis and data were collected using the flow cytometer.

### Immunofluorescence staining

After cells were fixed with 4% paraformaldehyde for 15 min, the primary podocytes were permeabilized with 0.1% Triton X-100 for 30 min at room temperature, and blocked with goat serum. Then, antibody (Santa Cruz Biotechnology, CA, U.S.A.) against cytokeratin 18 (CK18) was added and incubated at 4˚C overnight. After three washes with PBS, they were incubated with Cy3-labeled secondary antibody (Beyotime, Haimen, China) for 1 h at room temperature. Subsequently, DAPI was added for nuclei staining, and the cells were then examined under a fluorescence microscope.

### Hoechst staining

The cells were cultured in six-well plates (10^4^ cells/well) overnight. After 24 h of treatment with 5 ng/ml of TNF-α, the cells were fixed with 4% paraformaldehyde at room temperature for 1 h, washed with PBS, stained with 10 μg/ml Hoechst 33258 (Sigma–Aldrich, MO, U.S.A.) at 37˚C in the dark for about 10 min and then washed with PBS. Using nuclear staining, the apoptotic cells were identified by chromatin condensation by fluorescence microscopy (Olympus, Tokyo, Japan).

### Western blots

To extract protein, the cells were lysed with RIPA buffer. The total protein concentrations in whole-cell lysates were measured by the BCA Protein Assay. After heat denaturation, the cell lysates with 20 μg of protein and loading buffer were added into each well of SDS/PAGE. After electrophoresis, the proteins were transferred onto PVDF membranes, and blocked with 5% skim milk in TBST (TBS + Tween 20) for about 1.5 h at room temperature. Then the bands associated with the proteins of interest were incubated with primary antibodies against hnRNP K (Abcam, ab23644), c-FLIP (Abcam, ab6144), GSK3β (Abcam, ab93926), ERK (Abcam, ab17942), Casapse 3 (CST. sc-271759), Caspase 8 (catalog no. sc-6136), p-GSK3β (Abcam, ab75745), p-ERK (Abcam, ab50011), and GAPDH (Abcam, ab181602) at 4°C overnight. After three washes with TBST, the samples were incubated with horseradish peroxidase-conjugated secondary antibodies (catalog no. sc-2005; Santa Cruz Biotechnology, CA, U.S.A.) at room temperature for 1.5 h. Following the final wash, the immunoreactive bindings were visualized with an ECL detection kit (Amersham, GE Healthcare). GAPDH was chosen as the internal reference.

### Quantitative RT-PCR

Total RNA was extracted from podocytes with TRIzol (Invitrogen, MA, U.S.A.) according to the manufacturer’s instructions and was reverse transcribed into cDNA using a PrimeScript RT-PCR kit (TaKaRa, Dalian, China). RT-qPCR was conducted with the cDNA using an SYBR Green PCR kit (Roche Penzberg, Upper Bavaria, Germany). The relative expression was calculated using the comparative cycle threshold (2^−ΔΔC_T_^) method. The primers used were listed as follows: hnRNP K, forward 5′-CAATGGTGAATTTGGTAAACGCC-3′, reverse 5′- GTAGTCTGTACGGAGAGCCTTA-3′; GAPDH, forward 5′- ACTTTGTCAAGCTCATTTCC-3′, reverse 5′-TGCAGCGAACTTTATTGATG-3′. GAPDH was used as an internal control to calculate the relative transcript levels.

### Statistical analysis

Data were presented as the mean ± SD of three independent experiments. Two-tailed Student’s t-tests and one-way ANOVA were used for statistical analysis. All statistical analyses were conducted with SPSS 21.0 (SPSS Inc., Chicago, IL, U.S.A.) software. Statistical differences were considered significant at *P*<0.05.

## Results

### Isolation and characterization of primary podocytes

**Figure 1:**
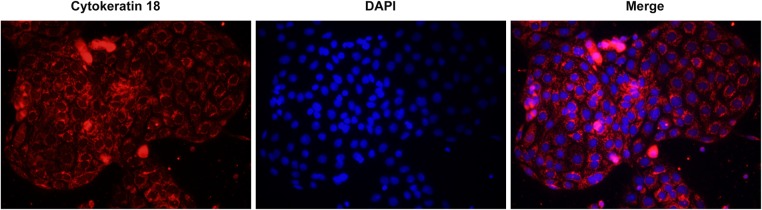
Expression of CK18 in primary podocytes Cells were fixed, permeabilized, and incubated with antibodies. CK18 was immunofluorescently stained red. The nuclei were stained blue with DAPI. Representative images are shown. Magnification = 400×.

We examined the expression of CK18, a marker of podocytes, in the isolated cells. Immunofluorescence staining showed that CK18 (Figure 1) was highly expressed in the cells, suggesting that podocytes were successfully isolated and characterized.

### TNF-α decreased podocyte growth

**Figure 2:**
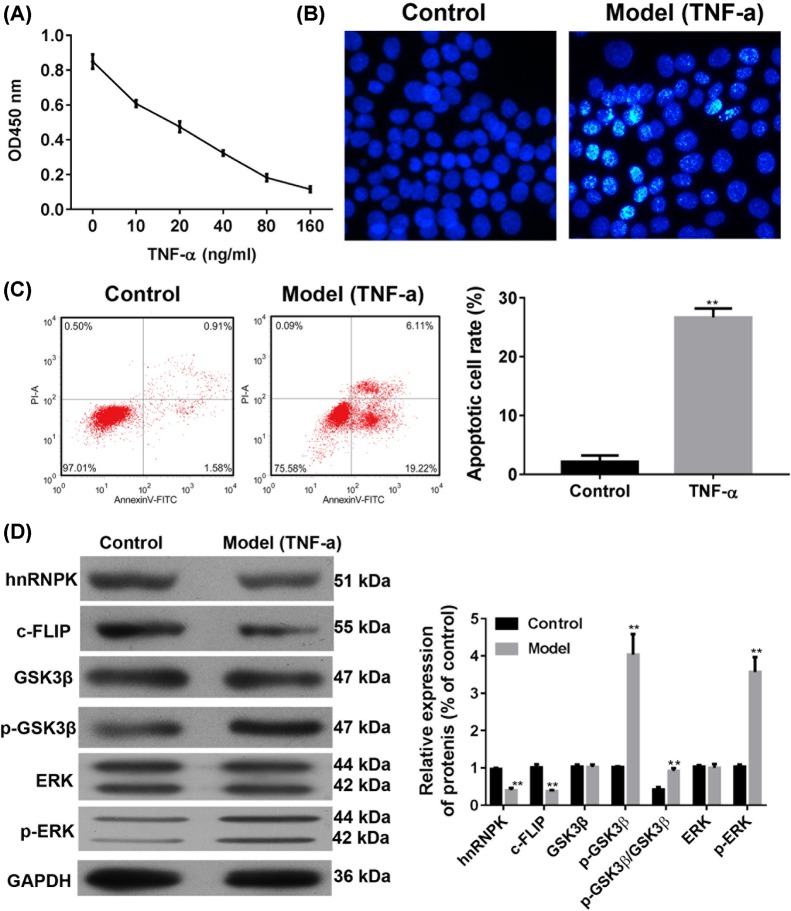
Effects of TNF-α on cell apoptosis and protein expressions (**A**) Primary podocytes were seeded in 96-well plates and were treated with TNF-α for 24 h, then the viability was measured by CCK8 assay. (**B**) After treatment with 5 ng/ml TNF-α for 24 h, apoptotic morphological changes in the primary cells were examined by Hoechest staining under fluorescence microscopy. (**C**) The apoptosis of primary podocytes were measured by flow cytometry. (**D**) Expressions of hnRNP K, c-FLIP, GSK3β, p-GSK3β, ERK, and p-ERK were analyzed by Western blot analysis. ^**^P<0.01, compared with the control group by two-tailed Student’s t test. Magnification = 400×.

We used the CCK8 assay to examine the effect of TNF-α on the viability of podocytes. After exposure to TNF-α for 24 h at the following concentrations: 10, 20, 40, 80, or 160 ng/ml, we observed a dose dependent decrease in cell viabilities (Figure 2A). Furthermore, we found that TNF-α inhibited cell growth at IC_50_ of 24.07 ng/ml, and that cell viability markedly decreased at 10 ng/ml, compared with the control group. To mimic apoptosis that occurs pathologically, we decided to use 5 ng/ml of TNF-α in subsequent experiments. As shown in Figure 2B,C, we found that 5 ng/ml of TNF-α significantly induced podocyte apoptosis. Flow cytometry analysis indicated the podocyte apoptosis rate increased from 2.5% in the control to approximately 25% in the experimental group. These results indicated that 5 ng/ml of TNF-α induced apoptosis in podocytes, and can be used to mimic the cell injury model *in vivo*.

Subsequently, we investigated the role of other regulators associated with TNF-α-induced apoptosis. As shown in Figure 2D, the densities of the immunoreactive bands of hnRNP K and c-FLIP were significantly decreased after 24-h exposure to TNF-α, as compared with those in the control group. In addition, the phosphorylated levels of GSK3β and ERK were notably increased. These data suggest that TNF-α contributed to apoptosis.

### hnRNP K knockdown enhanced TNF-α-induced apoptosis in podocytes

**Figure 3:**
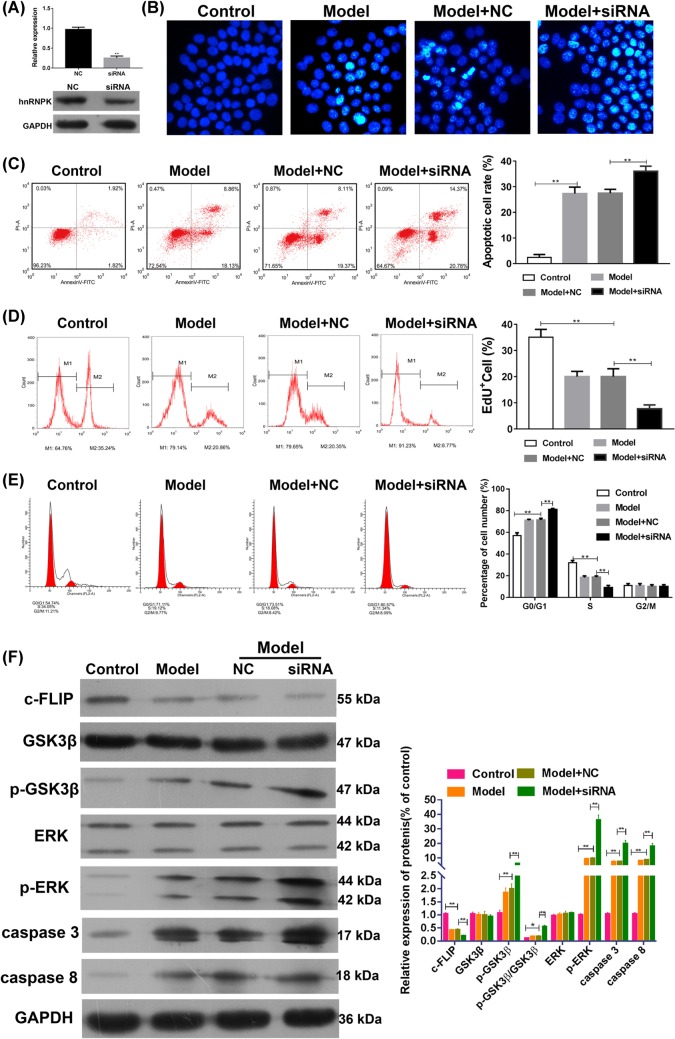
Effects of hnRNP K knockdown on cell apoptosis and protein expressions Primary podocytes were transfected with either siRNA against hnRNP K or NC. (**A**) Total RNA and proteins were isolated from the cells after transfection. RT-qPCR and Western blots were performed to detect hnRNP K mRNA expression in the primary podocytes. Primary podocytes with/without transfection were exposed to 5 ng/ml TNF-α or control for 24 h. (**B**) Apoptotic morphological changes in the primary cells were examined by Hoechest staining under fluorescence microscopy. By flow cytometry, the apoptosis (**C**), proliferation (**D**), and cell cycle distribution (**E**) of the podocytes were measured. (**F**) Western blotting was performed to detect the expressions of c-FLIP, GSK3β, p- GSK3β, ERK, p-ERK, caspase-3, and caspase-8 in the primary podocytes. Model indicated 5 ng/ml TNF-α exposure; ^**^P<0.01 by ANOVA followed by Bonferroni’s *post hoc* analysis. Magnification = 400×.

To investigate the role of hnRNP K in TNF- α-induced podocyte apoptosis, we knocked down the expression of hnRNP K using siRNA. As shown in Figure 3A, the expressions of hnRNP K at the transcriptional and translational levels were significantly decreased after transfection with the siRNA, indicating that hnRNP K knockdown was successful. After TNF-α treatment, we used Hoechest staining and found that the incidence of condensed DNA increased in the cells where hnRNP K expression was knocked down, compared with cells with NC transfection or without transfection, indicating that hnRNP K might play an inhibitory role in TNF-α-induced apoptosis (Figure 3B). This was confirmed by the flow cytometry assay, which revealed that hnRNP K knockdown increased TNF-α-induced apoptosis (Figure 3C) and correspondingly inhibited cell proliferation (Figure 3D), as compared with the groups with NC transfection or without transfection. In addition, cell cycle analysis showed that TNF-α treatment exhibited a significantly higher and lower frequency of cells at the G0/G1 and S phases, respectively. This was further enhanced by hnRNP K knockdown (Figure 3E). All these data suggest that hnRNP K inhibits the podocyte injury induced by TNF-α by promoting cell cycle arrest and inhibiting entry into S phase. Moreover, TNF-α might induce podocyte apoptosis by decreasing the expression of hnRNP K.

Subsequently, we observed the expression levels of other apoptosis regulators. As shown in Figure 3F, hnRNP K knockdown significantly enhanced the inhibitory effect of c-FLIP expression, as well as the stimulatory effect of GSK3β and ERK phosphorylation. Furthermore, the markers associated with apoptosis, caspase-3, and caspase-8, exhibited a significant increase in response to hnRNP K knockdown. These results suggest that TNF-α induces podocyte apoptosis by decreasing hnRNP K expression, and thus decreasing levels of c-FLIP, and increasing levels of GSK3β and ERK phosphorylation. In other words, hnRNP K is a target of TNF-α-induced apoptosis, and regulates the expressions of c-FLIP, GSK3β, and ERK phosphorylation.

### hnRNP K overexpression inhibited TNF-α-induced apoptosis in podocytes

**Figure 4:**
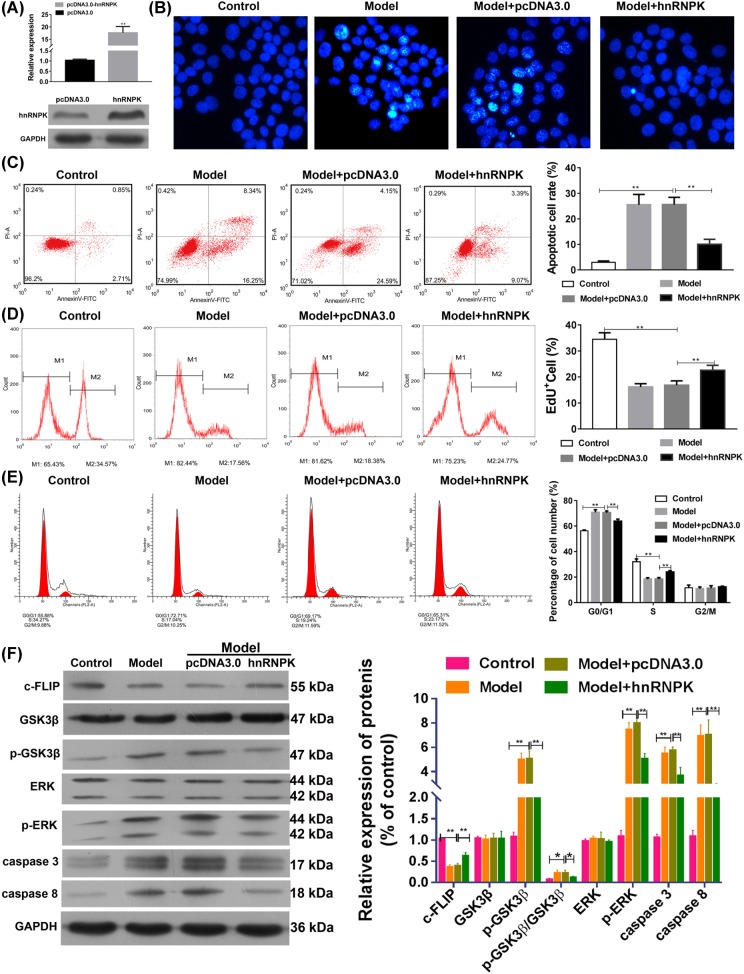
Effects of hnRNP overexpression on cell apoptosis and protein expressions Primary podocytes were transfected empty pcDNA3.0 vectors (pcDNA3.0) or pcDNA3.0 containing cDNA encoding hnRNP K. (**A**) Total RNA and proteins were isolated, then RT-qPCR and Western blotting were performed to detect hnRNP K mRNA and protein expressions, respectively, in the transfected primary podocytes. Primary podocytes with/without transfection were exposed to 5 ng/ml TNF-α or vehicle for 24 h. (**B**) Apoptotic morphological changes in the primary cells were examined by Hoechest staining under fluorescence microscopy. By flow cytometry, the apoptosis (**C**), proliferation (**D**), and cell cycle distribution (**E**) of the podocytes was measured. (**F**) Western blotting was performed to detect the expressions of c-FLIP, GSK3β, p- GSK3β, ERK, p-ERK, caspase-3, and caspase-8 in the primary podocytes. pcDNA3.0 indicated the primary podocytes received transfection of empty pcDNA3.0 vectors; pcDNA3.0-hnRNP K indicated the primary podocytes received transfection of pcDNA3.0 containing cDNA encoding hnRNP K. ^**^P<0.01, by ANOVA followed by Bonferroni’s *post hoc* analysis. Magnification = 400×.

To confirm the antiapoptotic effect of hnRNP K on podocytes exposed to TNF-α, we successfully transfected hnRNP K into podocytes. We observed an increase in mRNA and protein levels of hnRNP K (Figure 4A). In contrast to the effects of hnRNP K knockdown on cell viability, hnRNP K overexpression markedly decreased the frequency of condensed DNA and cell apoptosis induced by TNF-α (Figure 4B,C). Correspondingly, the cell proliferation inhibited by TNF-α was significantly increased (Figure 4D). In addition, the cell cycle distribution assay showed that hnRNP K overexpression significantly decreased and increased the incidences of G0/G1 phase and S phase, respectively, in the cells treated with TNF-α (Figure 4E). Western blots showed that TNF-α-treated cells overexpressing hnRNP K had significantly increased c-FLIP expression, and decreased GSK3β, ERK phosphorylation and caspase-3, caspase-8 expressions (Figure 4F). All these results confirm that the inhibitory effect of hnRNP K on TNF-α-induced apoptosis is mediated by its regulatory effects on the expressions of c-FLIP, and phosphorylation of GSK3β and ERK.

## Discussion

Proteinuria is the presence of excess protein in urine, and is a major healthcare problem affecting several hundred million people worldwide. As a well-known marker of kidney damage, proteinuria is closely associated with glomerulonephritis and end stage renal disease (ESRD) [15,16]. Typically, increased proteinuria is an indication of a decline in renal function, regardless of baseline estimated glomerular filtration rate (eGFR) [17]. Since podocytes are an essential component of the glomerular filtration barrier, any injury can result in proteinuria. Reducing proteinuria is often associated with beneficial effects on kidney damage [18], suggesting that protecting podocytes from injury might be a good treatment option against the kidney diseases associated with proteinuria. In the present study, we confirmed the apoptotic effect of TNF-α on podocytes. Furthermore, we demonstrated for the first time that hnRNP K contributes to TNF-α-induced apoptosis, in podocytes.

TNF-α has also been shown to be involved in renal disease, such as acute kidney injury. Numerous *in vivo* and *in vitro* studies have shown that exposure of glomerular cells to TNF-α induces glomerular dysfunctions similar to those observed in glomerulonephritis [19]. Furthermore, several studies suggest that TNF-α induces podocyte apoptosis [6,20]. Thus, TNF-α contributes to the initiation and development of glomerular injury, consistent with a previous report showing that it closely associates with ESRD [21].

As a master regulator of gene expression, hnRNP K has multiple functions in regulating cell proliferation, apoptosis, metastasis, and chemoresistance. Aberrant expression of hnRNP K can be induced by various effectors, such as epidermal growth factor (EGF) and heregulin-β1 [22,23]. However, limited studies exist that describe the impact of cytokines downregulating the protein. In the present study, we showed for the first time that TNF-α inhibited the expression of hnRNP K in podocytes, and consequently increased cell apoptosis. Moreover, we observed that overexpression of hnRNP K significantly suppressed the apoptotic effect induced by TNF-α. This is consistent with the widely reported antiapoptotic role of hnRNP K [24].

The antiapoptotic effect of hnRNP K has been primarily demonstrated by inducing the expression and activation of important oncogenes, such as *c-myc* and *c-SRC* [25]. In this study, we observed that the expression of c-FLIP was positively correlated with hnRNP K levels. Consistent with our observations, c-FLIP, an antiapoptotic protein, was highly expressed in tumor cells, and this high-level expression was significantly correlated with high-level hnRNP K expression [26]. The upregulation of c-FLIP was reported to occur transcriptionally via direct interaction with a poly(C) sequence in the FLIP promoter by hnRNP K/nucleolin complex [26]. Moreover, Quintavalle et al. found that c-FLIP can interfere with Gsk3β phosphorylation, resulting in the antiapoptotic effect [27]. However, work conducted by Gao et al. showed that hnRNP K inhibited Gsk3β phosphorylation, and subsequently increased the levels of c-FLIP expression by increasing stabilization [10]. It is possible that hnRNPK up-regulates the c-FLIP protein levels in cancer cells through other mechanisms [10]. In the present study, we did not establish the exact correlation between c-FLIP and Gsk3β phosphorylation, and this should be studied further.

A recent study demonstrated that ERK phosphorylation participates in the apoptosis of podocytes [28]. This is consistent with our observation that increased phosphorylated ERK coincided with increased podocyte apoptosis. Moreover, increased activation of ERK was accompanied by c-FLIP overexpression. Safa et al. showed that c-FLIP overexpression induced ERK activation, suggesting that ERK phosphorylation observed in this study might be mediated by c-FLIP [29]. In contrast, expression of c-FLIP protein has been reported to be regulated by the ERK pathway [30]. Future work needs to be done to determine the exact relationship between c-FLIP and ERK.

In podocyte apoptosis, the death receptor signaling pathway and activation of the caspase-8 (or caspase-10)–caspase-3 cascade are one of three major lethal signaling cascades []. In the pathway, pro-caspase-3 is cleaved and activated by caspase-8. The data in this study showed that the levels of caspase-8 and caspase-3 were significantly induced by TNF-α, and this is further mediated by hnRNP K. As a target of hnRNP K, c-FLIP is able to modulate activation of procaspase-8 and thereby prevent induction of apoptosis mediated by the death receptors [31,32]. All these support the notion that TNF-α induced caspase-8 and capase-3, as a result of reduced expression of hnRNP K.

In summary, we investigated the molecular details of TNF-α-induced apoptosis in podocytes. We found that in the podocyte model of TNF-α-induced apoptosis, hnRNP K was significantly decreased, which in turn decreased the expression of c-FLIP; whereas it increased GSK3β and ERK phosphorylation as well as caspase-8 and capase-3 activation. Although the correlation amongst increased GSK3β, ERK phosphorylation, and decreased c-FLIP expression was not investigated in this study, caspase-8 and caspase-3 induction is the net result of the correlation.

## Abbreviations

CK18: cytokeratin 18
ERK: extracellular signal-regulated kinase
ESRD: end stage renal disease
GSK3β: glycogen synthase kinase β
hnRNP K: heterogeneous nuclear ribonucleoprotein K
NC: negative control
PI: propidium iodide
TNF-α: tumor necrosis factor-α
TRAIL: TNF-related apoptosis-inducing ligand

## References

[1] Chen X., Qin Y., Zhou T. (2017) The potential role of retinoic acid receptor alpha on glomerulosclerosis in rats and podocytes injury is associated with the induction of MMP2 and MMP9. Acta Biochim. Biophys. Sin. (Shanghai) 49, 669–679 10.1093/abbs/gmx066 28645189

[2] Dai R., Lin Y., Liu H. (2015) A vital role for Angptl3 in the PAN-induced podocyte loss by affecting detachment and apoptosis in vitro. BMC Nephrol. 16, 38 10.1186/s12882-015-0034-4 25884163 PMC4383073

[3] Ryu M., Mulay S.R., Miosge N., Gross O. and Anders H.J. (2012) Tumour necrosis factor-alpha drives Alport glomerulosclerosis in mice by promoting podocyte apoptosis. J. Pathol. 226, 120–131 10.1002/path.2979 21953121

[4] Tharaux P.L. and Huber T.B. (2012) How many ways can a podocyte die? Semin. Nephrol. 32, 394–404 10.1016/j.semnephrol.2012.06.011 22958494

[5] Saito Y., Okamura M., Nakajima S. (2010) Suppression of nephrin expression by TNF-alpha via interfering with the cAMP-retinoic acid receptor pathway. Am. J. Physiol. Renal Physiol. 298, F1436–F1444 10.1152/ajprenal.00512.2009 20237236

[6] Guo Y., Song Z., Zhou M. (2017) Infiltrating macrophages in diabetic nephropathy promote podocytes apoptosis via TNF-alpha-ROS-p38MAPK pathway. Oncotarget 8, 53276–53287 28881810 10.18632/oncotarget.18394 PMC5581109

[7] Wu Q., Yang Z., Nie Y., Shi Y. and Fan D. (2014) Multi-drug resistance in cancer chemotherapeutics: mechanisms and lab approaches. Cancer Lett. 347, 159–166 10.1016/j.canlet.2014.03.013 24657660

[8] Abdo S., Lo C.S., Chenier I. (2013) Heterogeneous nuclear ribonucleoproteins F and K mediate insulin inhibition of renal angiotensinogen gene expression and prevention of hypertension and kidney injury in diabetic mice. Diabetologia 56, 1649–1660 10.1007/s00125-013-2910-4 23609310

[9] Gao R., Yu Y., Inoue A., Widodo N., Kaul S.C. and Wadhwa R. (2013) Heterogeneous nuclear ribonucleoprotein K (hnRNP-K) promotes tumor metastasis by induction of genes involved in extracellular matrix, cell movement, and angiogenesis. J. Biol. Chem. 288, 15046–15056 10.1074/jbc.M113.466136 23564449 PMC3663525

[10] Gao X., Feng J., He Y. (2016) hnRNPK inhibits GSK3beta Ser9 phosphorylation, thereby stabilizing c-FLIP and contributes to TRAIL resistance in H1299 lung adenocarcinoma cells. Sci. Rep. 6, 22999 10.1038/srep22999 26972480 PMC4789638

[11] Wang Z., Ge Y., Bao H., Dworkin L., Peng A. and Gong R. (2013) Redox-sensitive glycogen synthase kinase 3beta-directed control of mitochondrial permeability transition: rheostatic regulation of acute kidney injury. Free Radic. Biol. Med. 65, 849–858 10.1016/j.freeradbiomed.2013.08.169 23973862 PMC3859848

[12] Chen S., Cao W., Yue P., Hao C., Khuri F.R. and Sun S.Y. (2011) Celecoxib promotes c-FLIP degradation through Akt-independent inhibition of GSK3. Cancer Res. 71, 6270–6281 10.1158/0008-5472.CAN-11-0838 21868755 PMC3185138

[13] Xu W., Ge Y., Liu Z. and Gong R. (2014) Glycogen synthase kinase 3beta dictates podocyte motility and focal adhesion turnover by modulating paxillin activity: implications for the protective effect of low-dose lithium in podocytopathy. Am. J. Pathol. 184, 2742–2756 10.1016/j.ajpath.2014.06.027 25239564 PMC4188873

[14] Liu Y., Liang W., Yang Q. (2013) IQGAP1 mediates angiotensin II-induced apoptosis of podocytes via the ERK1/2 MAPK signaling pathway. Am. J. Nephrol. 38, 430–444 10.1159/000355970 24247724

[15] Jafar T.H., Stark P.C., Schmid C.H. (2001) Proteinuria as a modifiable risk factor for the progression of non-diabetic renal disease. Kidney Int. 60, 1131–1140 10.1046/j.1523-1755.2001.0600031131.x 11532109

[16] Matsushita K., van der Velde M. and (2010) Association of estimated glomerular filtration rate and albuminuria with all-cause and cardiovascular mortality in general population cohorts: a collaborative meta-analysis. Lancet 375, 2073–2081 10.1016/S0140-6736(10)60674-5 20483451 PMC3993088

[17] Turin T.C., James M., Ravani P. (2013) Proteinuria and rate of change in kidney function in a community-based population. J. Am. Soc. Nephrol. 24, 1661–1667 10.1681/ASN.2012111118 23833255 PMC3785273

[18] Vozmediano C., Rivera F., Lopez-Gomez J.M. and Hernandez D. (2012) Risk factors for renal failure in patients with lupus nephritis: data from the spanish registry of glomerulonephritis. Nephron Extra 2, 269–277 10.1159/000342719 23139689 PMC3493001

[19] Ishikawa K., May C.N., Gobe G., Langenberg C. and Bellomo R. (2010) Pathophysiology of septic acute kidney injury: a different view of tubular injury. Contrib. Nephrol. 165, 18–27 10.1159/000313740 20427951

[20] Pedigo C.E., Ducasa G.M., Leclercq F. (2016) Local TNF causes NFATc1-dependent cholesterol-mediated podocyte injury. J. Clin. Invest. 126, 3336–3350 10.1172/JCI85939 27482889 PMC5004940

[21] Singh K., Prasad K.N., Mishra P. (2015) Association of tumour necrosis factor-alpha polymorphism in patients with end stage renal disease. Nephrology (Carlton) 20, 387–391 10.1111/nep.12398 25589331

[22] Sugimasa H., Taniue K., Kurimoto A., Takeda Y., Kawasaki Y. and Akiyama T. (2015) Heterogeneous nuclear ribonucleoprotein K upregulates the kinetochore complex component NUF2 and promotes the tumorigenicity of colon cancer cells. Biochem. Biophys. Res. Commun. 459, 29–35 10.1016/j.bbrc.2015.02.043 25701787

[23] Barboro P., Ferrari N. and Balbi C. (2014) Emerging roles of heterogeneous nuclear ribonucleoprotein K (hnRNP K) in cancer progression. Cancer Lett. 352, 152–159 10.1016/j.canlet.2014.06.019 25016060

[24] Gallardo M., Hornbaker M.J., Zhang X., Hu P., Bueso-Ramos C. and Post S.M. (2016) Aberrant hnRNP K expression: all roads lead to cancer. Cell Cycle 15, 1552–1557 10.1080/15384101.2016.1164372 27049467 PMC4934053

[25] Hu C.E., Liu Y.C., Zhang H.D. and Huang G.J. (2014) The RNA-binding protein PCBP2 facilitates gastric carcinoma growth by targeting miR-34a. Biochem. Biophys. Res. Commun. 448, 437–442 10.1016/j.bbrc.2014.04.124 24796666

[26] Chen L.C., Chung I.C., Hsueh C. (2010) The antiapoptotic protein, FLIP, is regulated by heterogeneous nuclear ribonucleoprotein K and correlates with poor overall survival of nasopharyngeal carcinoma patients. Cell Death Differ. 17, 1463–1473 10.1038/cdd.2010.24 20224598

[27] Quintavalle C., Incoronato M., Puca L. (2010) c-FLIPL enhances anti-apoptotic Akt functions by modulation of Gsk3beta activity. Cell Death Differ. 17, 1908–1916 10.1038/cdd.2010.65 20508645

[28] Liu S., Ding J., Fan Q. and Zhang H. (2010) The activation of extracellular signal-regulated kinase is responsible for podocyte injury. Mol. Biol. Rep. 37, 2477–2484 10.1007/s11033-009-9761-6 19728154

[29] Safa A.R. (2012) c-FLIP, a master anti-apoptotic regulator. Exp. Oncol. 34, 176–184 23070002 PMC4817998

[30] Seidelin J.B., Coskun M., Vainer B., Riis L., Soendergaard C. and Nielsen O.H. (2013) ERK controls epithelial cell death receptor signalling and cellular FLICE-like inhibitory protein (c-FLIP) in ulcerative colitis. J. Mol. Med. 91, 839–849 10.1007/s00109-013-1003-7 23371318

[31] Shirley S. and Micheau O. (2013) Targeting c-FLIP in cancer. Cancer Lett. 332, 141–150 10.1016/j.canlet.2010.10.009 21071136

[32] Lavrik I.N. and Krammer P.H. (2012) Regulation of CD95/Fas signaling at the DISC. Cell Death Differ. 19, 36–41 10.1038/cdd.2011.155 22075988 PMC3252827

